# Male Sex is an Inherent Risk Factor for Basal Cell Carcinoma

**DOI:** 10.1155/2019/8304271

**Published:** 2019-10-20

**Authors:** Ioannis D. Bassukas, Athina Tatsioni

**Affiliations:** ^1^Department of Skin and Venereal Diseases, Faculty of Medicine, School of Health Sciences, University of Ioannina, Ioannina, Greece; ^2^Department of Internal Medicine, Faculty of Medicine, School of Health Sciences, University of Ioannina, Ioannina, Greece

## Abstract

Basal cell carcinoma (BCC) is more frequent among females <40 years old; however, it affects preferentially older males (>60 years old). In order to contribute to the study of the still largely unknown mechanisms that underlie this peculiar sex-dependent shift, we compared the kinetics of the increase of the age-specific BCC incidence rates (*R*) as a function of age in males and females. Studies reporting sex-stratified *R* were found using a PubMed search and male to female age-specific incidence rate ratios (RR) were calculated for each age-class as reported in each study and assigned to the mean of the corresponding age periods. Trends in age were assessed with Kendall's *τ* test and relationships between two variables by inverse variance method-weighed Loess and linear regression analysis.

Sixteen data sets were eligible and confirmed a significant shift in the male to female ratio (Kendall's *τ* = 0.530; *P* < 0.001). Moreover, the slope parameter *b* = 1.205 (SE = 0.014) of the best fit (*r*^2^ = 0.980) regression line resulting by plotting male vs. female age-specific incidence rates predicts a statistically significant (*P* = 0.001), constant, about 20% faster increase of *R* in males of all ages. Similar relationship are also evident for cutaneous squamous cell and Merkel cell carcinoma and, even more intriguing, for sums of all cancers (excluding BCC and SCC) in many different registries.

In conclusion, females are probably born with an inherently higher risk to develop BCC; however, also with a much slower increase rate of this risk as a function of age. Notably this observation seems to be not a BCC peculiarity. Because of its high incidence coupled with moderate morbidity and extremely low mortality rates, BCC may serve as a valuable, single-tumor paradigm to reproach the complex mechanisms that underline the interaction of age and sex in the pathogenesis of human malignancies.

## 1. Introduction

Basal cell carcinoma (BCC) is currently the most frequent single cancer type in individuals of European ancestry worldwide and a tumor that preferentially affects older males within the 6^th^ to 8^th^ decades of life [1]. The reasons why this cancer, being more frequent for females among individuals younger than about 40 years becomes later in life a disease with distinct androtropy are still elusive. Herein, in order to examine the increment kinetics of BCC incidence as a function of age, studies reporting age-specific incidence rates were identified from the literature and the patterns of age-dependency of age-specific BCC incidence rates between females and males were compared.

## 2. Material and Methods

Based on a PubMed literature search (February 15, 2018) from the initially 1151 papers localized with the search string “[(basal cell carcinoma) OR (skin cancer)] AND (age-specific incidence)” 181 studies reporting sex-stratified, age-specific incidence rates (*R*) of BCC were identified according to abstract content for full-text evaluation. The criteria for the final selection step of the studies to be included in the analysis are compiled in Table 1. To ensure sufficient number of age classes (“age-sensitivity”) only papers reporting BCC data in age classes ≤10 years and at least 2 age classes for patients younger than 40 years were included. Only papers were considered that presented data in Table form. In order to have sufficient cases representation in all age classes only those studies were considered that reported ≥250 disease cases (either “numbers of patients” or “numbers of tumors”). To balance for a confounding phototype effect, only studies reporting data from European countries or populations of European descent were included (Table 1). Finally, studies reporting either data of selected age ranges or certain anatomical locations, e.g. the nose, were excluded. Each reported *R*-value was assigned to the median of the corresponding age span. For the same age class of each study male to female *R* ratios (RR) were calculated and were assigned to the corresponding median age. Using sex ratios of BCC incidence rates for of each age class and each publication separately is anticipated to ameliorate possible genetic (and phototype) variability between the reference populations of the different studies (this approach is further commented in [2]). Approximate standard errors of RR were calculated based on the available sex-stratified “case numbers”. Employing SPSS (Chicago, IL, USA) age trends were assessed with Kendall's *τ* test and relationships between two variables by inverse variance-weighed Loess and linear regression analysis. Statistical significance was indicated by *P* value <0.05 (two-sided).

## 3. Results

**Figure 1:**
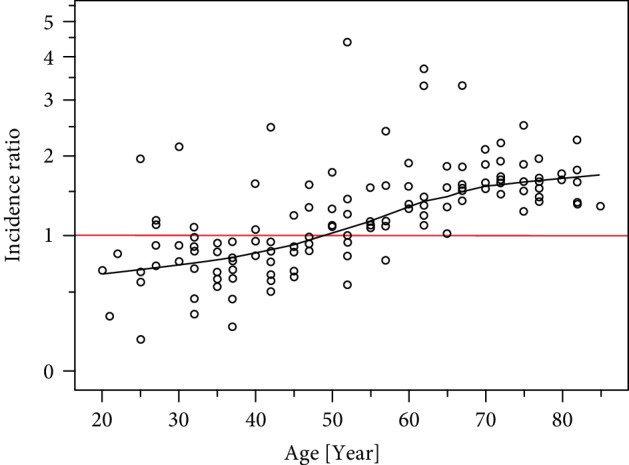
Male to female ratio of BCC incidence rates as a function of age. Each point represents ratio determination from paired data, i.e., for the same age period within each study. The best fit Loess line is shown (with Epanikov kernel at 50%). It crosses with the RR = 1 line of equal incidence rates of males and females (red line) at the age between 45 and 50 years.

**Figure 2:**
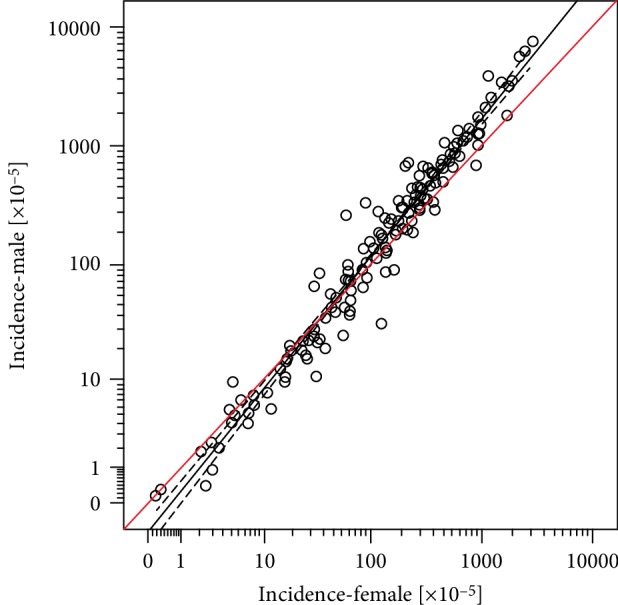
Age-specific BCC incidence rates (cases/100,000 population) of males as a function of the “twin” female data (same age range, same data set). Black line: best fit regression line (*r*^2^ = 0.980; dashed lines: 95% confidence intervals of predicted value). Red line: line of equal incidence rates of the two sexes (slope: *b*′ = 1.000).

Sixteen data sets (published between 1988 and 2014) were eligible (Table 2). In parallel to the known increase of *R* with age for both sexes (not shown), we confirmed a significant shift in the male to female ratio of the age-specific incidence rates as a function of age (Kendall's *τ* = 0.530; *P* < 0.001; Figure 1). For persons younger than ~45–50 years, *R* is lower and for those >50 years higher for males (significantly higher in the ages 60–80 years).  Figure 2 displays dyads of related male vs. female age-specific BCC incidence rates (same data set, same age). The slope parameter of the best fit (*r*^2^ = 0.980) regression line *b* = 1.205 (standard error: SE = 0.014) predicts a statistically significant about 20% faster *R* increment in males (*P* = 0.001). Notably the relationship of the increase rates of *R* between the two sexes seems to be constant and independent of age. In addition, the value of this increment is not significantly affected by the different coding of BCC cases in the different studies (Table 2; *P* = 0.074 for the comparison “number of patients” vs. “numbers of cases”).

## 4. Discussion

Age and sex are two major, nonmodifiable factors of the risk of an individual to be diagnosed with cancer [3]and together these two factors significantly modulate the incidence of the disease at the population level: Cancer is a disease of aging [4] and taking all age groups together a disease that is more frequent in males than in females; however, not in all ages [2, 5]. The well-known higher sum cancer incidence of males as a whole results from the fact that cancer is more frequent among males than females in those age groups with the highest disease risk, i.e., elderly people. Our present data compilation suggests that with respect to above epidemiologic characteristics, BCC behaves like an “average cancer”, i.e., might serve as a tumor prototype to study above relationships. The age-specific risk of BCC increases steadily with age for both sexes and by several degrees of magnitude from childhood to, at least up, to the 10^th^ decade of life [1]. In addition, as presently shown, sex substantially modifies the age-specific BCC risk in a dual fashion: At an age around leaving adolescence, females seem to be at a higher risk to develop BCC than males. However, on the other hand, at any age the risk to be diagnosed with BCC (*R*) per unit risk increases as a function of age (*a*) by an excess, but constant and age-independent factor of about 20% (increment factor: 1.205) significantly faster in males (*R*_*M*_) than in females (*R*_*F*_): [*dR*_*M*_/*da*]/*R*_*M*_ = 1.205 × [*dR*_*F*_/*da*]/*R*_*F*_(Figure 2). This complex age dependence of BCC disease risk results into higher BCC incidence rates for females until the age of about 40–50 years [6]and significantly lower ones in the >60 years age group (androtropism in the older).

Both biologic and behavioral factors have been proposed to explain above age dependency and sex differences in the incidence rates of cancer (oxidative stress, genome structure and gene expression, immunocompetence, connective tissue stability etc.) [2]. Particularly, the preferential use of tanning beds by younger females than males [7]has been incriminated as an added sex-specific photocarcinogenesis hazard to explain the higher skin cancer incidence of younger females [8]. Our present observation that the BCC risk increases as a function of age more steeply in post-adolescent males of all ages than females cannot be solely explained by differences in the patterns of exposure of the two sexes to skin-specific environmental carcinogens. On the one hand, the constant and independent of age proportionality of the specific BCC risk increment rates between the two sexes is more consistent with the hypothesis of an inherent, biologic factor underlying above relationship. On the other hand, the pattern of higher BCC risk among younger females and at the same time lower among older females compared to males of the same age is not a finding peculiar to this tumor type. Also the age-specific incidence rates of the cutaneous squamous cell carcinoma and of the Merkel cell carcinoma increase as a function of age more abruptly for males compared to females.[9] Even more intriguing, similar sex-characteristic evolution patterns of age specific cancer risk as a function of age are also apparent for the sum of all cancers (excluding keratinocyte skin cancer) in the U.S. population as previously noticed by Cook et al. [2] and is also confirmed by recalculating the last SEER data release (16.3% higher increment rate of the age specific cancer incidence rate of nonhispanic white males >15 years compared to females, calculated from the SEER Cancer Statistics Review, 1975–2016 data [10]; *p* = 0.959). Similar relationships between the increment rates of age-specific all-cancer incidence rates between males and females are also evident in data from the Australian population,[11] as well as in global compilations of cancer incidence rates (employing the GLOBOCAN data [12] set we calculated a 14.7% higher increment rate of the age-specific cancer incidence rate of males >15 years compared to females; *p* = 0.978). It is worth noting that sex seems to be an inherent, biologic feature of the mechanism that underlies fixing and accumulation of mutations in human cancers. In parallel to presently discussed differences in the modes of the increase of the age-specific incidence rates as a function of age the number of mutations per whole exom increases for a series of different nonsex-specific cancers at a significantly higher rate in males compared to females [13]. To our opinion these latter observations are also in line with the recently reviewed evidence that the keratinocyte skin cancer is a marker of a high cancer-risk phenotype [14].

The main limitations of the present study are literature-based design, nonuniform mode of reporting the cases in the included studies and scarcity of data reporting ‘extreme' age groups (patients <25 and patients >85 years old). Another limitation is the fusion of data reporting either numbers of patients or tumor in the present compilation. However, as already mentioned, a subgroup analysis of the male vs. female age-specific BCC incidence rates does not reveal significant differences between the two coding types of data report (patients vs. tumors numbers).

Age and sex are two main, nonmodifiable, constitutive factors of BCC susceptibility that interact in a characteristic mode to determine the evolution of BCC disease risk during life. We would like to suggest that because of its high incidence coupled with overall moderate morbidity and extremely low mortality rates [15] BCC may serve as a valuable, single‐tumor paradigm to reproach the complex mechanisms that underlie the interaction of age and sex in the pathogenesis of human malignancies.

## Figures and Tables

**Table 1:** Criteria for the selection of data sets.

Inclusion criteria	At least 2 subclasses for ages <40 years
	Patient / tumor numbers are available in age classes
	Age-specific data for the two sexes separately
	Incidences reported in age intervals ≤10 years
	≥250 patients/cases included
	Reference population: Study from a European country or population of European descent

Exclusion criteria	Restricted to selected age subgroups
	Focus on selected anatomical localizations

**Table 2:** Compilation of included data sets.

Data set	PMID	First author [^a^]	Publication year	Country	Follow up period	Coding	Age range^b^	Age classes	Age class mode^c^
1	7547221	Levi [16]	1988	Switzerland	1976–1985	Patient	(30–34)-(85+)	5 yr	N0–N4
2	2322501	Roberts [17]	1990	UK	1988	Patient	(25–29)-(85+)	5 yr	N0–N4
3	2312827	Chuang [18]	1990	USA	1976–1984	Patient	(0–14)-(85+)	10 yr	N5–[N+1]4
4	1954125	Coebergh [19]	1991	Netherlands	1975–1988	Patient	(0–14)-(85+)	5 yr	N0–N4
5	1985867	Magnus [20]	1991	Norway	1976–1982	Neoplasm	(−19)-(90+)	10 yr	N0–N9
6	11122025	Holme [21]	2000	UK	1998	Patient	(25–29)-(85+)	5 yr	N0–N4
7	11568742	Harris [22]	2001	USA	1985–1996	Patient	(<20)-(>80)	10 yr	N0–N9
8	14578155	Athas [23]	2003	USA	1977–1978	Neoplasm	(25–34)-(75+)	10 yr	N5–[N+1]4
9	14578155	Athas [23]	2003	USA	1998–1999	Neoplasm	(25–34)-(75+)	10 yr	N5–[N+1]4
10	14520447	Stang [24]	2003	Germany	1995–1999	Patient	(30–34)-(85+)	5 yr	N0–N4
11	17640064	Bath-Hextall [25]	2007	UK	1996–2003	Patient	(18–24)-(80+)	5 yr	N0–N4
12	17550552	Stang [26]	2007	Germany	1998–2003	Patient	(10–19)-(>80)	10 yr	N0–N9
13	18649084	Radespiel-Tröger [27]	2008	Germany	2001–2005	Patient	(0–29)-(80+)	10 yr	N0–N9
14	19796178	Bielsa [28]	2009	Spain	2006–2007	Neoplasm	(0–4)-(85+)	5 yr	N0–N4
15	19595266	Celic [29]	2009	Croatia	2003–2005	Neoplasm	(0–9)-(80+)	10 yr	N0–N9
16	24874476	Reinau [30]	2014	UK	2000–2011	Patient	(18–19)-(80+)	5 yr	N0–N4

^a^Reference number. ^b^Younger and oldest age group considered. ^c^*N*  = integer 0–9; for example for *N* = 4: N0–N9 corresponds to 40–49 and N5–[N+1]4 to 45–54.

## Data Availability

All used data are published and available in the literature.
